# Application of emerging technologies in ischemic stroke: from clinical study to basic research

**DOI:** 10.3389/fneur.2024.1400469

**Published:** 2024-06-10

**Authors:** Qiuyan Chen, Shuxia Zhang, Wenxiu Liu, Xiao Sun, Yun Luo, Xiaobo Sun

**Affiliations:** ^1^Institute of Medicinal Plant Development, Peking Union Medical College and Chinese Academy of Medical Sciences, Beijing, China; ^2^Beijing Key Laboratory of Innovative Drug Discovery of Traditional Chinese Medicine (Natural Medicine) and Translational Medicine, Beijing, China; ^3^Key Laboratory of Bioactive Substances and Resource Utilization of Chinese Herbal Medicine, Ministry of Education, Beijing, China

**Keywords:** ischemic stroke, medical imaging, disease phenotypes, mechanisms research, emerging technology, basic research, clinical study

## Abstract

Stroke is a primary cause of noncommunicable disease-related death and disability worldwide. The most common form, ischemic stroke, is increasing in incidence resulting in a significant burden on patients and society. Urgent action is thus needed to address preventable risk factors and improve treatment methods. This review examines emerging technologies used in the management of ischemic stroke, including neuroimaging, regenerative medicine, biology, and nanomedicine, highlighting their benefits, clinical applications, and limitations. Additionally, we suggest strategies for technological development for the prevention, diagnosis, and treatment of ischemic stroke.

## 1 Introduction

Stroke ranks globally as the second-leading cause of mortality and the third-leading cause of disability (1). Ischemic stroke (IS), the most common type, is increasing in both incidence and burden. Data from the Global Burden of Disease (GBD) indicate that the global incidence and mortality rates of IS have increased by 88 and 61%, respectively, between 1990 and 2019 (2). Empirical evidence demonstrates a lower incidence of stroke morbidity and mortality in high-income regions in contrast to their low-income counterparts (2), likely attributed to the presence of superior healthcare services and more advanced medical technology. Furthermore, a noteworthy decline of 36.0% (range, 31.0–42.0%) in age-standardized mortality rates and disability-adjusted life years due to stroke was observed worldwide between 1990 and 2019 (2), underscoring a potential association with advances in medical technology. These advances have markedly improved the diagnosis and treatment of stroke, leading to better patient recovery. This review discusses IS from the perspectives of neuroimaging, regenerative medicine, molecular biology, and nanomedicine, summarizing the applicability of various techniques and the challenges faced in clinical translation.

Advances in neuroimaging have facilitated early stroke recognition and revascularization. For instance, non-contrast computed tomography (NCCT) scans enable the swift exclusion of hemorrhagic stroke, while CT angiography effectively detects large vessel occlusions and provides valuable insight into the underlying causes of stroke. CT perfusion can assess cerebral blood flow and define the ischemic core, Lastly, a comprehensive process involving advanced magnetic resonance imaging (MRI) techniques provides immediate information essential for making treatment decisions for ISs. However, it is imperative to acknowledge that each imaging modality possesses inherent limitations, and making well-informed decisions, while considering these limitations, is pivotal in optimizing the efficacy of thrombolysis and thrombectomy in the larger patient population.

Tissue engineering and regenerative medicine (TERM) is an emerging interdisciplinary field that combines the use of cells, scaffolds, and growth factors to regenerate or replace damaged or diseased tissues. While TERM has been under development for over three decades, it still remains in its nascent stages, with a variety of unresolved issues. Furthermore, the complex processes controlling the formation of new tissues or organs *in vivo* using tissue-engineered materials, along with the fate and transformation of these materials, are critical concerns in this dynamically evolving domain. Consequently, the clinical translation of techniques such as stem cell transplantation and 3D bioprinting remains a challenge that requires resolution.

In recent years, new technologies, such as single-cell RNA sequencing (scRNA-seq) and spatial transcriptomics, have emerged as powerful tools for elucidating the mechanisms underlying stroke pathogenesis, scRNA-seq enables high-resolution identification of cell-specific markers and can thus be used to study cellular heterogeneity, identifying the major clusters of cells in the brain, the expression patterns of specific genes, and the functions of cell subclusters in pathways associated with IS. Spatial transcriptomics can analyze the transcriptomes of cells or tissues in relation to spatial information. Therefore, a combination of scRNA-seq with spatial transcriptomics can solve the problem of loss of cellular spatial information during the pre-processing of scRNA-seq samples. Combining both methods can expand our understanding of brain tissue in the presence of stroke and can thus enable precise treatment. However, the use of both scRNA-seq and spatial transcriptomics is still in its infancy in neuroscience, especially in the field of stroke.

Small-molecule chemical probes represent a valuable tool for studying the biological functions of proteins and their relevance as therapeutic targets. This technique has the advantages of structural diversity, ease of use, high selectivity, and good biocompatibility. Certain probes can be used to enable non-invasive, real-time monitoring and high-throughput screening within living organisms, Consequently, these probes have found application in various medical domains, including pathological research, drug screening, and the clinical diagnosis of brain diseases (3, 4). In the context of stroke development and progression, alterations occur in the contents or activities of various substances in the brain, such as reactive oxygen species (ROS) and enzymes. The development of fluorescent probes utilizing these substances as biomarkers has promising efficacy for detection and monitoring in the context of stroke. Nevertheless, it is imperative to take into account the constraints posed by the blood-brain barrier (BBB) and the depth of penetration for both the excitation and emission light pertaining to these fluorescent probes.

The field of nanomedicine aims to use the properties and physical characteristics of nanomaterials for the diagnosis and treatment of diseases at the molecular level. Nanoparticles (NPs) play a pivotal role in improving the pharmacological and pharmacokinetic profiles of conventional drugs, and can be used as carriers for targeted drug delivery (5). Due to their small sizes and specific surface modifications, many drug-loaded NPs can cross the BBB, thus enhancing drug bioavailability. Moreover, these NPs can enhance the properties of drugs, including solubility, stability, and half-life, leading to reduced drug doses and increased dosing intervals (6). Furthermore, certain NPs can reduce the levels of ROS and chelate iron, mitigating secondary stroke damage caused by oxidative stress and iron overload (7, 8). Nevertheless, despite significant advances in nanomedicine research, several challenges remain unresolved before these technologies can be integrated into clinical practice.

This review primarily delineates the application and challenges of emerging technologies in the context of IS within traditional treatment paradigms. The article provides a comprehensive synthesis of viable improvement measures proposed by previous researchers to address identified shortcomings. Structurally, the content is divided into two distinct sections, namely, clinical and pre-clinical. Each emerging technique is discussed individually, with the overarching goal of providing valuable insights to aid clinicians and researchers in their decision-making processes.

## 2 Clinical study

### 2.1 Clinical techniques for characterizing disease phenotypes

#### 2.1.1 MRI

Magnetic resonance imaging (MRI) is indeed a valuable tool in the diagnosis and assessment of stroke, and it utilizes various imaging sequences to provide a comprehensive view of the brain and associated lesions. These different sequences assist clinicians in a better understanding of the nature and extent of brain abnormalities, including those associated with stroke (9). Unlike CT, it is both non-invasive and radiation-free with a higher resolution in soft tissue, allowing effective imaging of lesions of the nervous system (10). In patients with acute ischemic stroke (AIS), due to the sudden onset of the condition and small window for treatment (11), accurate localization of ischemic lesions and the determination of ischemic severity are key to the treatment of AIS. Standard sequences [such as T_1_-weighted, T_2_-weighted, and fluid-attenuated inversion recovery (FLAIR)] are relatively insensitive for detection of hyperacute ischemia. Fortunately, diffusion-weighted imaging (DWI, a new MRI techniques) is highly sensitive and can detect the presence of ischemia at an early stage. This advantage is due to the imaging principle of DWI, whereby it can quantitatively measure the Brownian motion of water molecules in the extracellular matrix with the apparent diffusion coefficient (ADC).

For AIS with an unknown time of onset, MRI can identify patients who are in the time window for thrombolysis therapy (4.5 h) by the mismatch between DWI and FLAIR in the ischemic area (12). A DWI-positive lesion without a corresponding change in FLAIR suggests that patients with this imaging feature are likely to benefit from thrombolysis (12). Beyond its role in clinical diagnosis, MRI can also be used for the prediction of cognitive impairment subsequent to IS. A study by Peng et al. (13) revealed that the combination of a serum biomarker for axonal damage, namely, neurofilament light chain (NfL), with MRI imaging markers, such as the infarction volume and white matter hyperintensities (WMH), enhances the prognosis of patient cognitive outcomes upon discharge and can thus facilitate tailored treatment strategies for IS. Ospel et al. (14) elaborated further on this issue in their research on the utility of DWI and non-contrast computed tomography (NCCT) for both qualitative and quantitative assessment of stroke parameters, such as infarct volume and the extent of white and gray matter damage. Their research found that confinement of the infarct to the gray matter, sparing of corticospinal tracts, and scattered infarct structure observed on 24 h NCCT and DWI were highly predictive of good 90-day clinical outcome, independent of the total infarct volume.

Although MRI offers many clinical benefits, its use is limited by its high cost and its inapplicability to patients with metal implants (e.g., prostheses, cardiac stents, MR-incompatible pacemakers, and implantable cardioverter defibrillators). In addition, MRI can show blood flow obstruction in the vascular lumen, although it cannot visualize the thrombus itself. Furthermore, because calcifications in the blood vessel walls do not contain protons, it is difficult to distinguish the thrombus from the potential atherosclerotic plaque (15), which may affect treatment.

To improve access to MRI, a recent publication described a low-field, portable MRI (pMRI) that was cost-effective, user-friendly, and safer for use in individuals with internal metal implants. This innovation reduced artifacts caused by such implants and circumvented the need for specialized shielding due to its operation at a low magnetic field strength of 0.064 T. Consequently, would facilitate dynamic bedside assessment of IS (16). In addition, the development of high-resolution magnetic resonance imaging (HR-MRI) has made submillimeter evaluation of intracranial arterial walls possible (17). Zhang et al. (18) found that T1-weighted HR-MRI could display intracranial occlusive thrombi directly. For patients with acute intracranial artery occlusion undergoing screening by magnetic resonance angiography (MRA), the detection rate of luminal thrombi by HR-MRI reached 96.4%, including the discovery of stroke-unrelated thrombi in 7.4% of patients, a novel finding that demonstrates the heterogenous applications of HR-MRI for intracranial occlusion.

#### 2.1.2 MRA

Magnetic resonance angiography (MRA) is highly sensitive to carotid occlusion and is thus commonly used for angiography during acute or subacute cerebral infarction (19). According to the different imaging principles, MRA can be divided into time-of-flight-MRA (TOF-MRA) and contrast-enhanced-MRA (CE-MRA). TOF-MRA is routinely employed for the evaluation of major blood vessels, including the intracranial and carotid arteries, and is well-suited for non-invasive vascular imaging. CE-MRA has the advantages of fast imaging and high anatomical coverage. Nevertheless, it requires the use of gadolinium-based contrast agents, which carry the risk of causing nephrogenic systemic fibrosis (20). However, this risk can be bypassed by TOF-MRA, and the use of TOF-MRA in 3D imaging mode is thus ideal for intracranial vascular imaging (21). However, the vascular signals in TOF-MRA are dependent on the direction and velocity of the blood, which may overestimate the degree of stenosis. To increase diagnostic accuracy, studies have used 3D high-resolution black-blood MRI combined with TOF-MRA for vascular wall imaging. Black-blood MRI is an imaging modality employed to attenuate high-intensity signals and is commonly used for the delineation of anatomical structures such as blood vessel walls and clots. Through the suppression of blood-related signals, this technique can identify components such as lipid deposits and blood clots on the vascular wall, compensating for the limited depiction of vascular wall complexities typically observed in TOF-MRA (17). By combining the two, more realistic, accurate, and complete images of blood vessels can be obtained.

Cerebral aneurysm is a risk factor for stroke, and patients undergoing surgery for intracranial aneurysms require long-term follow-up. Digital subtraction angiography (DSA) is considered the gold standard for such postoperative follow-up due to its high spatial resolution. However, it is an invasive procedure, and repeat surgeries increase the risk, reducing its suitability for follow-up. MRA is a non-invasive imaging method that avoids the risks associated with radiation exposure and complications from contrast agents. However, due to magnetization and radiofrequency shielding, its visualization of blood flow in the treated segment of the artery remains limited. To overcome the limitations of TOF-MRA, silent MRA has emerged as a new follow-up tool. A study by Li et al. (22) compared the roles of silent and 3D-TOF MRA in carotid artery atherosclerosis. The results showed that silent MRA provided excellent image quality of the carotid siphon and demonstrated higher consistency with DSA. Silent MRA can also provide a more realistic and accurate reflection of blood flow while offering a quieter imaging environment, and can thus be used to monitor treated intracranial aneurysms during follow-up (23).

#### 2.1.3 MRS

Magnetic resonance spectroscopy (MRS) is an imaging technique that utilizes MRI technology to obtain information on metabolic spectra in biological tissues, providing both qualitative and quantitative data on tissue metabolism, including the concentrations, ratios, and chemical shifts of various organic and inorganic metabolites. Oxygen deprivation caused by IS compromises the metabolic functions of brain cells in the ischemic region. This hypoxic state triggers anaerobic glycolysis, leading to the accumulation of lactate and reduced pH. An increased lactate peak can be observed in MRS images. Furthermore, MRS can assess the level of lactate metabolism as the degree of acidosis varies in the ischemic core and the penumbral area. N-acetylaspartate (NAA) is found in neurons and is considered a marker of neuronal health. Therefore, during IS, a decrease in the NAA peak is typically observed (24). In addition to individual metabolite concentrations, MRS can also provide information about the ratios between different metabolites, which can be used to evaluate the metabolic state and function of brain tissue. For instance, a reduction in the ratio of NAA to creatine (Cr) can reflect neuronal death or damage (25).

The clinical application of MRS is, however, limited by its low spatiotemporal resolution. Li et al. (26) proposed a novel ^1^H-MRSI technology termed SPICE (SPectroscopic Imaging by exploiting spatiospectral CorrElation). which can yield simultaneous 3D whole-brain images of important neurometabolites such as NAA and lactate with a spatial resolution of 2 × 3 × 3 mm^3^ within 8 min, and can effectively distinguish the ischemic penumbra of AIS.

#### 2.1.4 CT

Non-contrast CT (NCCT) is the preferred diagnostic modality for the initial assessment of patients presenting with suspected stroke. It exhibits a high degree of sensitivity in the detection of intracranial hemorrhage, thereby enabling the differentiation between ischemic and hemorrhagic stroke (27). NCCT offers the advantages of cost-effectiveness and rapid availability, although it does involve the use of ionizing radiation. Nevertheless, in clinical practice, NCCT is administered at a safe radiation dose, with an average effective dose of 2 mSV for adults, while doses ranging from 0.9 to 4.0 mSV have been reported in the medical literature (28). After the use of NCCT to exclude hemorrhagic stroke, it is necessary to use both CT angiography (CTA) and CT perfusion (CTP) to evaluate cerebrovascular blood flow and delineate the core infarction area. This process is intricate and pivotal. Some researchers have suggested that NCCT and CTA information can be derived directly from CTP images to streamline the scanning process and reduce radiation exposure. This proposal is based on the principle that CTP inherently encompasses a richer dataset than the other two imaging techniques (29). Nevertheless, it is imperative to underscore that this does not imply the complete obsolescence of NCCT. In certain cases, parenchymal defects near the border may exhibit less hypodensity on reference NCCT scans and risk being misidentified as normal tissue.

NCCT exhibits relatively low sensitivity in the early detection of acute ischemic tissue, particularly within the first 3 h following stroke onset when observable changes are often subtle or virtually absent (30). Consequently, NCCT may be unable to discriminate effectively between stroke and stroke-mimicking conditions, such as migraine with aura and focal epilepsy. The integration of artificial intelligence (AI) for automated recognition holds significant promise in enhancing diagnostic accuracy (31), consequently, it is likely that the convergence of AI and medical imaging will provide substantial clinical benefits in the future.

#### 2.1.5 CTA

Computed tomography angiography (CTA) is the most frequently employed follow-up diagnostic modality after head CT imaging, and represents an alternative to conventional digital subtraction angiography (DSA). CTA has several advantages, including (1) rapidity, (2) greater availability in the general community compared with MRA, and (3) reduced dependence on hemodynamic effects compared with MRA. On account of its 100% sensitivity and specificity in detecting large vessel occlusions, it is mainly used to identify large intracranial vessel occlusions and carotid and vertebral artery disease (32). Its diagnostic efficacy extends to enhanced identification of major blood vessels implicated in ischemic events, thus expediting the diagnosis of ischemic cerebrovascular disease within the narrow therapeutic window of 2 h post-onset. The prognostic significance of collateral circulation in patients with AIS is now widely acknowledged. While it is recognized that perfusion techniques such as computed tomography perfusion (CTP) can provide information on collateral circulation, they fall short in direct visualization. In contrast, CTA techniques, including single-phase CTA (sCTA) and multiphase CTA (mCTA), excel in the identification of occluded points and the visualization of collateral filling (33). mCTA includes three acquisition stages with a commendable temporal resolution, allowing a more detailed and reliable assessment of collateral vessels. Specifically, the first stage replicates the single sCTA stage with coverage from the aortic arch to the vertex. The latter two stages cover the skull from the base to the apex, and are mainly used for the visualization of intracranial vessels (34). Several studies have shown that mCTA has greater sensitivity and accuracy for the assessment of vessel occlusion and collateral circulation assessment than sCTA, mainly due to its ability to specifically detect small vessel occlusions and reduced influence by the timing of image acquisition (35–37). Moreover, CTA-source images have significant advantages over NCCT in the prediction of the final infarct size, especially in terms of collateral circulation and clot burden length (38). Conventionally, stroke management protocols recommend the administration of tissue plasminogen activator (t-PA) before CTA, and the CTA-Alberta Stroke Program Early CT Score (ASPECTS) can serve as a prognostic tool to predict the therapeutic efficacy of intravascular interventions in patients with stroke (39).

Regrettably, the utilization of iodine contrast media (CM) remains necessary for CTA, despite the potential risk of contrast renal fibrosis (40). Therefore, it is recommended to minimize the use of CM for patients at risk of acute kidney injury. However, reduced CM volume may affect the image quality. Thus, a balance between ensuring optimal image clarity and minimizing the harm caused by contrast agents is necessary. Lowering the tube potential can increase iodine attenuation, which can reduce the amount of CM required. Nonetheless, this measure also leads to increased image noise, necessitating a higher tube current for compensation. To address this challenge, Higashigaito et al. (41) designed algorithms and proposed personalized small-capacity CM schemes with automatic tube voltage selection for different individuals. A relationship between the tube voltage and the iodine decay curve has been observed, permitting the determination of an optimized CM scheme. The results of the study demonstrated that the final modified CM protocol was effective in all 61 patients (100%) in achieving the requisite image quality while reducing the overall CM dosage.

#### 2.1.6 CTP

Computed tomography perfusion (CTP) is a CT technique used to measure the perfusion of cerebral tissue. CTP involves the intravenous injection of a tracer followed by a CT scan to capture the time-density curve (TDC) as it traverses the region of interest. Post-processing software is then used to derive parameters such as cerebral blood flow (CBF), cerebral blood volume (CBV), and the mean transit time (MTT) of the tracer. Image reconstruction techniques are then utilized to generate a pseudo-color map.

CTP has numerous applications in AIS. Firstly, it plays a crucial role in assessing the extent of ischemia, analysis of the collateral circulation, and delineation of the ischemic penumbra. In CTP, the ischemic core is typically characterized by tissue showing reduced CBF and CBV (CBF/CBV matching). Salvageable regions of the ischemic penumbra, identified as tissue with low CBF but high or preserved CBV (CBF/CBV mismatch), can be distinguished from the ischemic core by determination of the CBF, CBV, and MTT (42). The primary goal of endovascular therapy is improvement in blood perfusion and timely saving of the agonal penumbra brain tissue, although hemorrhagic transformation (HT) is worrisome. The risk of hemorrhagic transformation can be predicted based on the perfusion parameters to determine whether patients can benefit from vascular recanalization. Studies have demonstrated the efficacy of CTP in predicting the risk of HT. The preoperative CBF in the HT area after mechanical thrombectomy was found to be lower than that in the cerebral infarction area, which was defined as a 30% reduction in CBF relative to the contralateral hemisphere in CTP (43, 44). Secondly, CTP allows the quantification of blood flow kinetics per unit volume, significantly enhancing the accuracy of clinical predictions regarding infarct volumes and focal infarctions (45, 46).

Differences in research methodology may lead to bias in the determination of the ischemic core by CTP. This variability is attributed to (1) the post-processing algorithm of CTP, (2) differences in research methodology, and (3) implementations of receiver operating characteristic (ROC) curve analysis (47–49). Consequently, the standardization of CTP protocols in stroke assessment is extremely important. The use of fully automated CTP post-processing software facilitates the standardized visualization of CTP data through a user-friendly color coding system. Nevertheless, the post-processing of CTP data is complex and subject to variation. Additionally, its predictive function may introduce bias due to the use of predetermined algorithms and thresholds. This bias can involve the mismatch between the actual CTP parameters and the assumed linear statistical model. To address this issue, Kuang et al. (45) proposed a deep machine learning (ML) model without thresholds, utilizing a random forest (RF) classifier to map multiple CTP parameters and temporal variables for each voxel to a statistical infarct probability model at the voxel level. This approach was found to be more accurate in the prediction of infarct volume and volumetric differences than existing methods.

#### 2.1.7 OCT

Optical coherence tomography (OCT) scanning represents a novel imaging modality after CT and MRI, offering minimally invasive and high-resolution capabilities. Its underlying principle is based on the variability in light-scattering properties of tissue components. The sample beam encounters scattering at interfaces between tissue structures with differing refractive indices, which are then combined with the reference beam to generate a 2D image of the tissue structure via spectral interference analysis (50). Key determinants influencing its clinical and research utility include axial resolution, transverse resolution, detection sensitivity, and measurement speed. Standard OCT typically shows average axial and transverse resolutions of 10 μm and 20 μm, respectively, leading to its description as “intravital microscopy.” In cardiovascular medicine, OCT is used to determine the type of vascular thrombosis, identify thin-cap fibroatheromas, and detect superficial calcifications, thereby aiding in the selection of stent implantation types and device placement (51). The US Food and Drug Administration (FDA) approved the use of intravascular OCT for diagnosing and treating cardiovascular diseases in 2010. OCT can rapidly and accurately identify the cause of the condition, primarily by assessing the degree of stenosis in the vertebral basilar artery, as approximately one-third of IS cases occur in the vertebral basilar artery system, and patients with vertebral basilar artery stenosis ≥50% have a higher risk of IS (52). Additionally, OCT can show the level of brain perfusion by distinguishing between moving particles and stationary tissue without the need for contrast agents. Nevertheless, there are certain limitations to the use of OCT for assessing intracranial vessels, especially when bends in intracranial arteries might restrict the advancement of the imaging catheter. Repeated attempts to pass through these curved intracranial arteries could result in catheter rupture and vascular damage (50). To address this, optimization of OCT imaging catheters could involve the use of smaller more flexible catheters.

Optical coherence tomography angiography (OCTA) is a new imaging technique based on OCT. OCTA uses motion-contrast imaging to obtain high-resolution information on volumetric blood flow, thus generating angiographic images without the use of dye in a matter of seconds (53). A recent study has shown the potential of OCTA in stroke monitoring by examining retinal microvascular parameters, revealing marked rarefaction of the retinal capillaries in both the macular and optic disc vascular plexus in patients with stroke (54). In addition, in terms of neural structures, OCTA showed that patients with stroke have thinner ganglion cell complex thickness (GCCt) and retinal nerve fiber layer thickness (RNFLt) (55). In summary, these observations suggest that OCTA parameters can provide supplementary insight into microvascular changes associated with stroke. However, further investigations are necessary to determine its utility as a reliable predictive marker.

However, OCTA has limited ability to penetrate tissue, resulting in poor imaging depth which has prevented its full substitution for traditional imaging methods. To address this limitation, Hou developed a high-power broadband p-doped near-infrared quantum dot broad-area light emitter (NIR-QD-BLE). This is characterized by a “J”-shaped ridge waveguide device structure and was able to overcome the challenge of trade-offs between the spectral width and output power, providing a promising foundational light source for near-infrared incoherent optical systems, especially OCT systems (56).

#### 2.1.8 Positron emission tomography/single-photon emission computerized tomography

Positron emission tomography (PET) is a nuclear medicine imaging modality that has had a significant impact on stroke research. PET can assess pathophysiological changes caused by ischemia, measure physiological parameters, and map the locations of molecular markers. Oxygen-15 positron emission tomography (^15^O-PET) is the gold standard for the detection of the ischemic penumbra (57), defined by low CBF (less than 20 mL/100 g/min), an increased oxygen extraction fraction (OEF), and an almost steady cerebral metabolic rate of oxygen (CMRO_2_) (58). The ability of PET to identify the penumbra within 48 h of stroke onset enables a timely understanding of the viability of the living tissue. This information is valuable for the development of personalized stroke treatment and aids in prognostic assessments of the disease. Kim et al. (59) used ^18^F fluorodeoxyglucose PET (^18^F-FDG-PET) to predict the risk of IS in patients with tumors. They found that arterial inflammation indicated by high FDG uptake in carotid plaque may be an independent predictive factor for IS.

Single-photon emission computed tomography (SPECT) resembles PET in its utilization of tracer molecules for circulatory imaging. Li et al. (60) used SPECT to identify a tracer targeting riboflavin transporter-3 (RFVT3), namely ^131^I-RFLA, which could be used as a novel biomarker for appropriate intervention in early stroke under the guidance of SPECT. In addition, it could also be used to assess the levels of atherosclerotic plaque.

In contemporary medical imaging, the standalone use of PET has been largely replaced by PET/CT. The latter not only provides biochemical and metabolic information but also captures details of anatomical structures. However, these advantages come at the cost of increased radiation exposure. Exploring an alternative such as the amalgamation of PET with MRI may be a viable option, as MRI does not rely on ionizing radiation. Additionally, encouraging the elimination of radionuclides can be achieved in patients through increased water intake and diuresis.

### 2.2 Clinical methods for disease treatment and mechanism research

#### 2.2.1 Metabolomics

Metabolomics has emerged after the development of genomics and proteomics and allows the qualitative and quantitative assessment of small-molecule metabolites. Cerebral ischemia induces alterations, both regional and systemic, in metabolism, impacting metabolic pathways involved in cellular energy and vital functions. Consequently, the identification of biomarkers associated with stroke progression is important for both clinical diagnostics and the exploration of underlying disease mechanisms.

**Figure 1:**
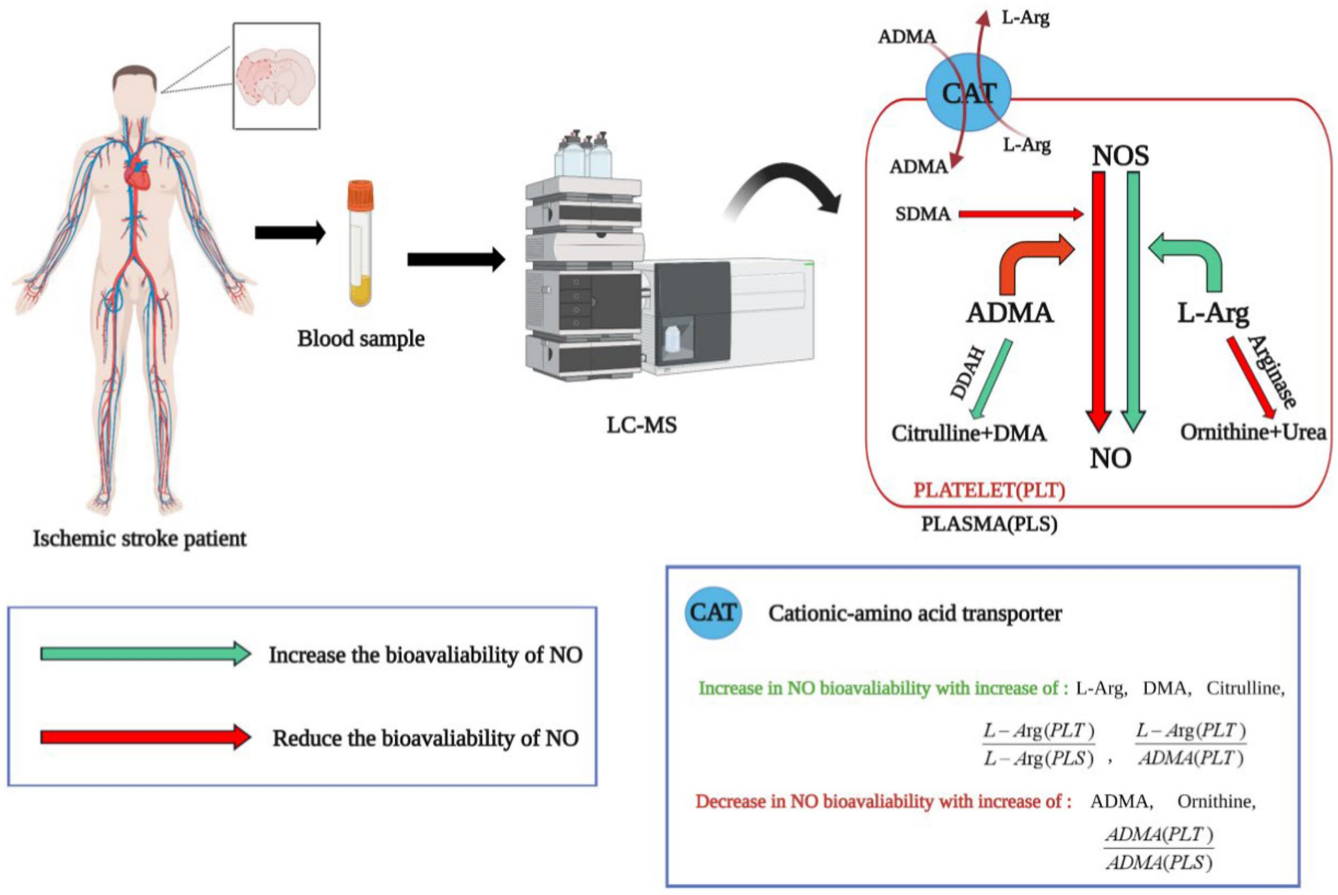
The application of targeted metabolomics in identifying biomarkers for ischemic stroke. NOS, nitric oxide synthase; L-Arg, L-Arginine; SDMA, symmetric dimethylarginine; ADMA, asymmetric dimethylarginine; DMA, dimethylamine; DDAH, dimethylarginine dimethylaminohydrolase. Arg is a direct synthetic substrate of NO, ADMA is an inhibitor of NOS and can be decomposed into citrulline and dimethylamine by DDAH. The value of L-Arg/ADMA reflects the bioavailability of NOS. The ratio of ADMA in platelets to plasma [ADMA (PLT)/ADMA (PLS)] represents the bioavailability of NOS inhibitors. The green arrows represent processes that increase NO bioavailability. The red arrow represents the process of reducing NO bioavailability. Through targeted metabolomics analysis, it was found that the characteristics of non-cardiac stroke patients include reduced bioavailability of nitric oxide (NO) in platelets. This is a new perspective in the field of ischemic stroke, suggesting that NO synthase in platelets could be a potential therapeutic target for ischemic stroke.

In addition to conventional IS biomarkers, such as sugars, lipids, amino acids, and folic acid (61). Tiedt et al. (62) found that four metabolites, namely, symmetric dimethylarginine (SDMA), asymmetric dimethylarginine (ADMA), pregnenolone sulfate, and adenosine were more effective in recognizing AIS and stroke mimics; these could be evaluated using non-targeted metabolomics, which was found to be superior to conventional multi-mode cranial CT. Specifically, these metabolites are associated with the regulation of vascular tone, inflammation, or stress. Thus, this metabolite set could serve as a valuable adjunct to CT imaging in emergency scenarios where MRI is unavailable, indicating its potential in clinical applications. Compared with non-targeted metabolomics, targeted metabolomics enables efficient quantitative analysis within a relatively brief timeframe. Bladowski et al. (63) used targeted metabolomics to investigate the potential association between the pathogenesis of non-cardiogenic stroke and alterations in nitric oxide (NO) homeostasis in platelets or plasma. The researchers monitored the ADMA ratio in platelets and plasma, serving as an indicator of the bioavailability of nitric oxide synthase (NOS) inhibitors, and observed increased levels of NOS inhibitors in platelets in patients with non-cardiac stroke. These increases were associated with increased platelet adhesion and aggregation, contributing to thrombosis formation. The findings suggested that modulation of platelet NO bioavailability could be used as a novel therapeutic target (Figure 1). Li et al. (64) investigated the use of metabolomics to differentiate between cardiogenic embolism and cerebral ischemia attributed to atherosclerosis. The study used ultra-high performance liquid chromatography-quadrupole time-of-flight mass spectrometry (UPLC-QTOF-MS) to identify six potential biomarkers for predicting the origin of thrombi. Subsequently, they assessed the predictive efficacy of these markers using a machine-learning model. It was found that these six metabolites exhibited robust discriminative potential between the two types of embolism and could serve as promising indicators for anticipating AIS induced by major blood vessel occlusion, thus having significant implications for the elucidation of AIS pathogenesis and enhancing secondary prevention strategies.

While the utilization of metabolomics in stroke research is still in its early stages compared to other metabolic disorders, there is merit in promoting the integration of this technology with stroke studies. This approach offers a novel perspective that enhances our understanding of the disease. Some metabolites identified in these studies provide microscopic insights into the pathology and address the limitations of earlier research, thereby facilitating more precise guidance for individualized treatment strategies following thrombectomy in different types of stroke.

#### 2.2.2 Stem cell transplantation

Stem cells (SCs) are characterized by high levels of self-renewal and the potential for multidirectional differentiation. In recent years, SC transplantation has shown great potential in IS treatment. SCs from different sources with different differentiation potentials are selected; these then undergo directed migration to sites of brain injury under the control of chemokines. This leads to the replenishment of damaged nerve cells, together with the generation of new blood vessels, inhibition of apoptosis, and the repair of neuronal function, thus alleviating neurological deficits and promoting recovery from IS (65). Currently, various types of SCs such as embryonic stem cells (ESCs), induced pluripotent stem cells, neural stem cells, and mesenchymal stem cells (MSCs) have been used to treat IS. Among them, MSCs are the most commonly used, as these have the advantages of easy culture and weak immunogenicity. MSCs can differentiate not only into cells of connective and muscle tissue but also into non-mesodermal cells, especially neurons and glial cells. When cultured under specific conditions, MSCs can form fully functional units called organoids, leading to the repair of damaged nerves (66). The immunomodulatory properties of MSCs and their secretion of bioactive factors have good therapeutic potential in stroke. MSC transplantation can, on the one hand, increase the expression of anti-inflammatory factors, such as interleukin-4 (IL-4), interleukin-10 (IL-10), and tumor necrosis factor β (TNF-β). On the other hand, the cells can inhibit the expression of pro-inflammatory factors, including interleukin-1 (IL-1), interferon γ (IFN-γ), tumor necrosis factor α (TNF-α), and membrane cofactor protein-1 (MCP-1) (67, 68). In addition to stimulating neurogenesis and regulating immunity, the most important neuroprotective effects of MSCs are activation of paracrine pathways and the production of extracellular vesicles (EVs). EVs share the same membrane orientation as cells, specifically, in the presence of lipids and transmembrane proteins on their extracellular surfaces that encase cytoplasmic components, including proteins and nucleic acids. The therapeutic effects of EVs in cerebral ischemia may be related to the regulation of many processes, including the induction of neurogenesis, activation of angiogenesis, inhibition of apoptosis, regulation of the immune response, and cellular reprogramming, showing similar effects to MSCs (69). More importantly, EV therapy has several advantages over the direct implantation of MSCs, namely, (1) lower immunogenicity, (2) better ability to cross the BBB, and (3) a higher body surface area ratio and greater ability to target tissues (see Table 1).

**Table 1:** Emerging technologies employed in clinical research.

	Classification	Advantage	Disadvantage	Application
Techniques used to diagnose diseases	MRI/(DWI)	High resolution of soft tissue, no radiation damage, multiple imaging parameters	Longer imaging time, more expensive, prohibition of patients with metal implants	Early cerebral ischemia, small ischemic lesion, posterior fossa lesions
	MRA	Rapid imaging, high-coverage, high sensibility to carotid artery occlusion	Contrast nephropathy (CE-MRA), overestimated degree of stenosis (TOF-MRA)	Angiography of acute, subacute or chronic cerebral infarction, follow-up after cerebral aneurysm
	MRS	Non-invasive, be able to quantitatively analyze the metabolism of tissues and organs	Long collection time, low spatial resolution, narrow coverage	Hypoxic ischemic encephalopathy (HIE), acute stroke
	CT	High sensibility to bleeding, be able to exclude cerebral hemorrhage, fast and low price	Radiation, poor discrimination of soft tissue	Exclude hemorrhagic stroke
	CTA	Non-invasive, cheap, be able to display the details of blood vessels	Potential risks of contrast media, contraindications for patients with renal insufficiency	Determination of the degree of coronary artery stenosis and the great artery occlusion
	CTP	Fast, high time resolution, provides information on multiple parameters: perfusion, blood volume, blood flow, etc	Cumbersome post-processing, motion artifacts, adverse reactions of contrast agents	judgement of the core area and penumbra, guide thrombolytic therapy
	OCT	Minimally invasive, fast, high resolution imaging	Weak penetration and shallow imaging depth	Monitor IS and guide intracranial vascular therapy
	PET/SPECT	Whole body imaging, high sensitivity, molecular level imaging	Expensive, radioactive radiation (PET), long scanning time (SPECT)	The gold standard for detecting ischemic penumbra
The technology used for mechanism research	Metabolomics	Changes in metabolite levels are easier to detect, the analysis methods are simpler, high-throughput analysis, non-invasive	High dynamic fluctuation, poor repeatability, influenced by factors such as medication, diet, and other variables	Provide biomarkers for early diagnosis of IS, provide improved prognostic assessments and enabling better evaluation of drug responses
	Stem Cell Transplantation	Almost comprehensively promote IS patients with neurological function repair, potential long-term therapeutic effects	The direction of stem cell differentiation is uncontrollable, cells transplanted into the brain have a lower survival rate, expensive	Nerve function repair after stroke
	Vagus nerve stimulation	Non-pharmacological treatments, safe	Risk of implantation surgery, infection and vagus nerve injury	Chronic functional recovery period patients after stroke
	Brain-computer interface	promotes neural plasticity	risk of infection, expensive equipment	improve the quality of life of patients with motor and language impairment

Currently, intravenous administration is the most commonly used method for delivering MSCs in clinical practice for the treatment of IS. However, an increasing body of research has demonstrated that the most significant efficacy of MSCs is associated with the extracellular vesicles secreted by the cells rather than their direct replacement of neurons (70, 71). Indeed, it has been found that most of the grafted cells are physically trapped at the precapillary level, with only a few reaching their targets and surviving for extended periods (72), Previous studies have shown that intravenous delivery of exosome-rich EVs produced by MSCs produces therapeutic effects similar to those of MSCs in animal models (73, 74), mainly manifest in promoting nerve/blood vessel regeneration and regulating immunity (75–77). EVs also have protective effects in *in vitro* models (78). In addition, on the one hand, EVs can indirectly regulate the expression of certain target genes by targeting specific miRNAs to up-or down-regulate the expression of miRNAs, thereby regulating IS-related pathological processes (79, 80). On the other hand, the presence of miRNA biomarkers in EVs after IS can be determined, enhancing our understanding of IS-triggered neuropathological processes (81). In summary, a large number of preclinical studies have revealed the effects of stem cell-derived EVs on IS (Table 2).

**Table 2:** Applications of stem cell-derived EVs in IS.

Reference	Animals	Stem cells/mode of application	EVs dose per animal	Effects
Gregorius et al. (82)	C57BL6/J mice	MSC/intravenous	EVs released by 2 × 10^6^ cells	Promote angiogenesis and nerve repair
Tian et al. (83)	C57BL6/J mice	ReNcell VM (ReN) cells/intravenous	100 μg	Antiinflammatory effect
Li et al. (84)	C57/BL6 mice	Inducible pluripotent stem cell (iPSC)/intravenous	10^9^ particles	The antisenescence ability on the BBB and antiinflammatory effect
Li et al. (85)	SD rat	MSC/intravenous	1 × 10^10^ particles	Attenuating BBB permeability
Zhang et al. (86)	C57BL/6	iPSC/intravenous	80 μg	Regulate intestinal microbial composition and biodiversity
Li et al. (87)	C57BL/6	MSC/intravenous	EVs released by 2 × 10^6^ cells	Enhance autophagy, improve bacterial clearance of macrophages, and prevent post-stroke pneumonia
Barzegar et al. (88)	C57BL/6	MSC/intraperitoneal	EVs released by 5 × 10^5^ cells	Maintain BBB integrity, ischemic hemisphere viability and perfusion

The application of stem cell-derived EVs, especially MSC-EVs, provides a means of enhancing the treatment of IS. At present, EVs have better safety profiles as they can avoid the possibility of malignant transformation by stem cells and pulmonary embolism events caused by the intravenous injection of stem cells. At the same time, EVs have the advantages of circulatory stability, low toxicity, avoidance of the first-pass effect, and good BBB permeability. However, their use is usually limited by the very low yields of conventional 2D cell culture systems, necessitating the scaling up of production for clinical applications. Culturing stem cells in 3D environments not only increases the efficiency of stem cell differentiation but also promotes the release of therapeutic EVs (89). Although stem-cell-derived EV therapies may represent a novel clinically viable cell-free paradigm, the basic research in this area is still in its early stages, although it has the potential to become a breakthrough in the future treatment of stroke and thus warranting in-depth study.

#### 2.2.3 Vagus nerve stimulation

Vagus nerve stimulation (VNS) is a neuromodulatory technique that delivers electrical signals to the vagus nerve. Implantable VNS (iVNS) was approved by the FDA in 2021 for the treatment of IS (90). The main mechanisms by which VNS exerts its therapeutic effects include (1) the indication of anti-inflammatory effects by inhibiting the production of pro-inflammatory cytokines, (2) inhibition of oxidative damage caused by glutamate release, (3) up-regulation of brain growth differentiation factor 11 (GDF11) involved in vascular remodeling, and (4) enhancement of neuroplasticity (91, 92). These have been verified in experimental models. In addition, several clinical studies have investigated the efficacy of iVNS on functional deficits of the upper limbs in patients with stroke. Dawson et al. (93) conducted a clinical trial on patients with moderate to severe upper-limb impairment. The patients were randomly divided into an iVNS+ rehabilitation group and a sham stimulation + rehabilitation group. After 90 days of outpatient treatment, more participants in the iVNS group than in the sham-stimulation group had a clinically meaningful response on the FMA-UE score, and the iVNS group also showed significant increases in the WMFT functional score. Kimberley et al. (94) conducted a study in patients with functional deficits in the arm after chronic stroke. After 6 weeks of treatment, the group receiving iVNS showed a significant increase in the FMA-UE scores compared with the control group, while after 90 days of treatment, the clinically meaningful response rate of FMA-UE was 88% with iVNS, compared with 33% in the control group.

The most serious complications of iVNS include infection and vocal cord paralysis due to vagus nerve injury (95), Furthermore, it is invasive, and equipment and surgical costs may be limiting factors for some patients. The device also requires regular maintenance, including battery replacement.

#### 2.2.4 Brain-computer interface

Brain-computer interface (BCI) represents a direct communication channel between the brain and external devices, which can convert brain signals into computational commands to control external devices. It can be used to restore motor function in limbs after stroke and thus has promising clinical significance for patients (96). BCI-based systems have been broadly classified as invasive and non-invasive systems based on the methods used to measure brain activity. At present, most BCIs used for stroke rehabilitation have utilized non-invasive methods such as EEG or MEG to receive brain signals (97). The key feature of non-invasive BCI is its safety, as it do not require surgically implanting electrodes into the brain. The advantage lies in avoiding the expensive and risky surgery, while also reducing the potential health risks. The second step of the BCI process usually involves the filtering and processing of the received brain signals to allow interpretation. Common techniques include sensorimotor rhythms and cortical slow potentials, followed by the use of signals such as a Fourier processor for translation of the signals into specific commands to be executed by the machine (98). The final BCI step generates responses for the programmed device, such as basic movement patterns, to improve the patient’s quality of life (99).

In April 2024, Meng et al. (100) conducted a clinical trial exploring the effectiveness and safety of using BCIs for upper extremity function recovery training in patients with IS. The results showed that on the basis of traditional rehabilitation training, BCI rehabilitation training could further improve patients’ upper extremity motor function. A total of 296 patients were enrolled in the study, with the average increase in the FMA-UE score after one month being 13.17 points for the BCI group and 9.83 points for the control group. Additionally, BCI technology is not used in isolation but is often combined with other rehabilitation techniques to enhance rehabilitation outcomes. For instance, the combination of brain-machine interface technology with repetitive transcranial magnetic stimulation (rTMS) can significantly reduce training time and improve the quality of detectable brain signals (101).

In summary, the application of BCI in the field of IS is constantly developing and improving, offering more opportunities for patient rehabilitation. Future research will continue to explore the potential and limitations of BCI technology, hoping to bring good news to more patients.

**Figure 2:**
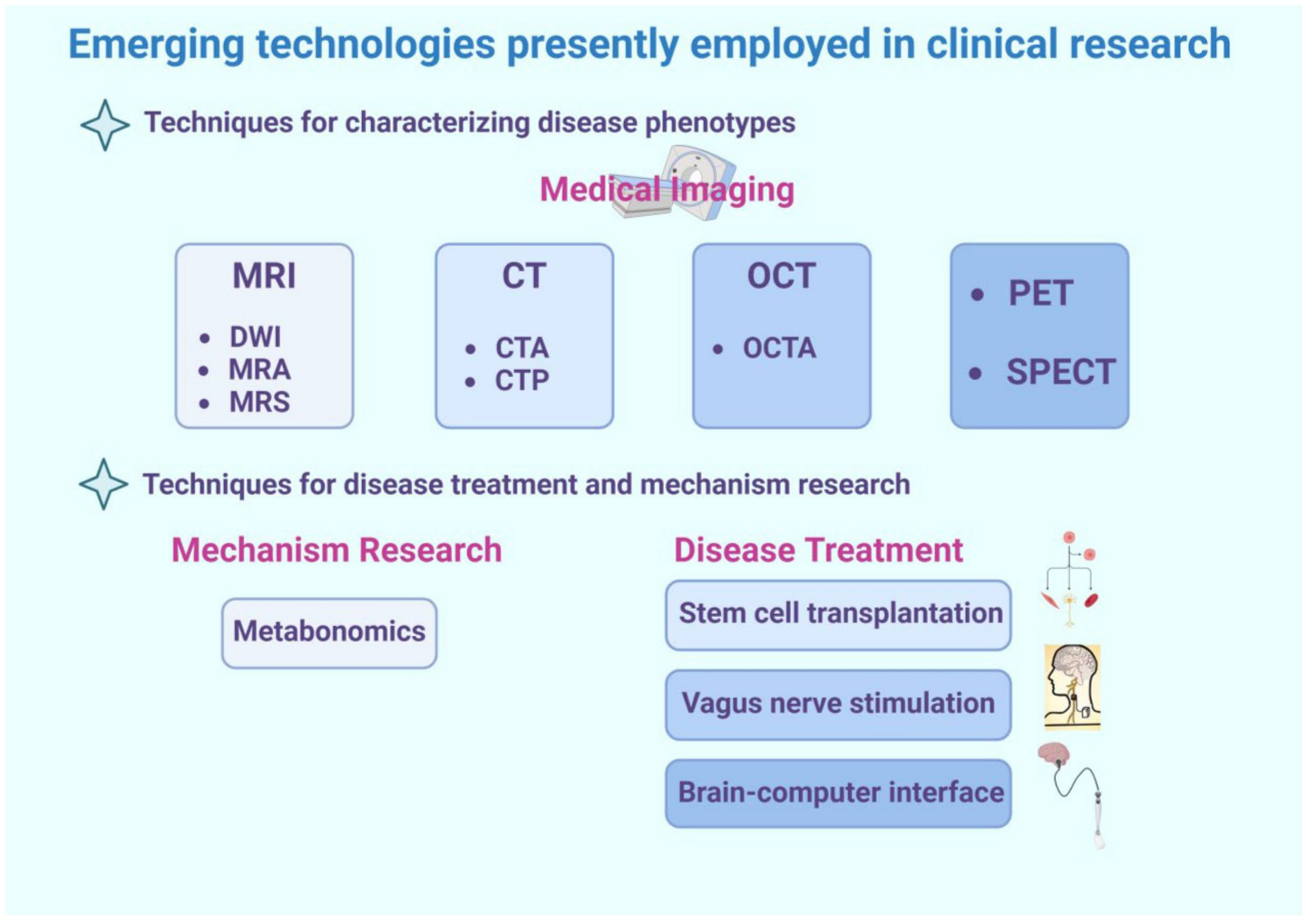
This is a graphical summary containing the emerging technologies covered in this review that are currently used in clinical research. The emerging technologies for clinical research are divided into two main categories, one of which is used for diagnosing diseases. This review primarily introduces some medical imaging techniques, such as magnetic resonance imaging (including various sequences such as DWI, MRA, MRS), CT (including some special types of CT such as CTA and CTP), optical coherence tomography (including its derivative technology OCTA), as well as PET and SPECT technologies. The second part includes technologies used for disease treatment and mechanism study, such as metabolomics, often used for disease mechanism study, and stem cell transplantation, vagus nerve stimulation, brain-machine interface technology, frequently used for disease treatment.

To assist readers in comprehending the clinical technologies involved in this review, refer to Figure 2.

## 3 Basic research

### 3.1 Technologies for diagnosis and treatment of disease

#### 3.1.1 Laser Doppler blood flow imaging

In the domain of laser flow monitoring, laser Doppler imaging (LDI) stands out as a real-time, minimally invasive, and highly sensitive technique for monitoring microcirculatory blood flow. It encompasses two modalities, namely, contact single-point blood flow detection and non-contact scanning imaging. The choice between these modes is contingent on various factors, including the experimental setup, the size and location of the target area, and the desired spatial resolution. Contact single-point blood flow detection allows in-depth measurements in specific areas, while non-contact scanning imaging covers the broader perspective of evaluation of blood flow patterns in larger tissue areas.

LDI is a valuable tool for the real-time monitoring of changes in cerebral blood flow (CBF) during middle cerebral artery occlusion (MCAO) surgery in small animals, facilitating the precise achievement of surgical objectives and assessments of the model success. Kaplan et al. (102) conducted a study utilizing LDI for the continuous and accurate measurement of to continuously and accurately measurement of the time threshold for recovery from reversible damage in a rat MCAO model. The results provided essential insights into the efficacy and safety of recirculating drug interventions beyond the recovery threshold. The study reported that prolongation of drug intervention beyond this threshold was not only ineffective in promoting recovery but also increased the risk of bleeding. These findings have significant implications for the clinical application of drugs in similar contexts. In a study by Wang et al. (103), LDI, in conjunction with 2D laser speckle contrast imaging, was used to assess the success of a mouse MCAO model. This approach yielded valuable insights, enabling a comprehensive evaluation of the usefulness of the model in stroke research. Chan et al. (104) employed multipoint laser Doppler to measure changes in the middle cerebral artery and perfusion in the collateral circulation in spontaneously hypertensive rats (SHR). A potential correlation was observed between increased damage in SHR and poor collateral circulation emphasizing the crucial role of robust collateral circulation in mitigating damage induced by cerebral ischemia, particularly in hypertensive diseases. Cuccione et al. (105) utilized multi-site laser Doppler imaging to evaluate collateral circulation after cerebral ischemia in mice. When combined with acute MRI, this approach enhanced the accuracy of stroke prognosis prediction.

Before the introduction of LDI, the assessment of MCAO models in China relied largely on subjective judgment derived from animal neurological deficit scores. This method was susceptible to subjectivity and prone to yielding false negative results. The introduction of LDI has effectively addressed this issue, contributing significantly to stroke research. Nonetheless, relative to laser speckle technology, LDI still has certain limitations in terms of convenience and functionality, as detailed in Table 3.

**Table 3:** Emerging technologies employed in basic research.

	Classification	Advantage	Disadvantage	Scope of application
Techniques used to diagnose and treat diseases	Laser Doppler blood flow imaging	Real-time, minimally invasive, high sensitivity	Operation is more troublesome than laser speckle imaging, small measuring area	Provide biomarkers for early diagnosis of IS
	Laser speckle flow imaging	Two-dimensional blood flow imaging, larger area imaging, high spatial and temporal resolution	Shallow imaging depth	Guide the construction of MCAO model, skin surface blood flow imaging
	Chemical probes	High sensitivity and selectivity, multi-target imaging, real time imaging	Limitation of imaging depth	Monitored the pathological status of IS and visualized the degree of vascular lesions
	Nanotechnology	Be able to enhance drug targeting and bioavailability, prolong the half-life of drug, increase brain delivery efficiency	Inadequate safety	Cerebral drug delivery, molecule imaging
The technology used for mechanism research	Single-cell RNA-sequencing	Provides a panoramic view of the composition, distribution, and function of cell subpopulations in a microenvironment, be able to analyze cell heterogeneity	Loss of cellular spatial information	Reveal the pathological mechanism of IS and discover new potential drug therapeutic targets
	Spatial transcriptomic	Cell heterogeneity was analyzed while spatial location information was provided	Low resolution, expensive	Used as a supplement to single-cell sequencing technology to solve the problem of loss of spatial information
	3D bioprinting	More realistic and complex 3D *in vitro* disease models	Speed and cost, material selection limitations	Limb orthotic device, surgical mould, scaffolds for stem cell transplantation
	16S rRNA sequencing	Be able to enhance drug targeting and bioavailability, prolong the half-life of drug, increase brain delivery efficiency	Inadequate safety	Cerebral drug delivery, molecule imaging

#### 3.1.2 Laser speckle flow imaging

Laser speckle imaging (LSI) is an intravital detection technique that has emerged in recent years. It can achieve rapid and significant spatial and temporal resolution in two-dimensional blood-flow imaging. Therefore, LSI can be used to map real-time monitoring of CBF. In basic research, LSI is often used to evaluate the construction of MCAO models. Li et al. (106) found that LSI allowed real-time observation of CBF in rats before, during, and after MCAO surgery, which could not only guide the construction of MCAO models but also predict the infarct volume. Liu et al. (107) combined LSI with visible-light optical coherence tomography (Vis-OCT) in the study of mouse ischemic models. LSI can detect CBF in real-time and guide Vis-OCT imaging, while Vis-OCT provides depth-resolved angiography and oxygen saturation (sO_2_) measurements.

Although LSI has the advantages of high spatial and temporal resolution and allows observations over a large field of view, it can only be used for superficial tissue imaging at present due to limitations in its imaging depth. How to perform deeper vascular imaging represents an important direction for future development.

#### 3.1.3 Chemical probes

Fluorescent probes are molecules that alter their fluorescence emission when combined with certain events, chemical reactions, or changes in their immediate environment. They have a wide range of applications in imaging, including *in vivo* imaging, tumor detection, monitoring of drug delivery, tracking of molecules *in vivo*, and super-resolution imaging (108–113). Most fluorescent probes can be classified according to their mechanism of diagnosing contrast media, namely, probes that are specific, non-specific, or able to be activated. Non-specific fluorescent probes are usually fluorescent dyes that take advantage of their differential distribution in healthy and diseased tissues to achieve contrast and can be used to assess changes in vascular permeability and perfusion in IS (114). Specific fluorescent probes can achieve higher target-to-background ratios, can be used to label key protein pathways, enzymes, and biomarkers in IS, and can locate tissues showing differential expression (115). Activated probes emit a fluorescent signal after enzymatic hydrolysis, and can thus be used as a tool to detect enzyme activity (116).

Near-infrared fluorescence imaging has the advantages of high sensitivity, high spatial and temporal resolution, and direct real-time manipulation. It can be used to visualize the extent of IS *in vivo* by the preparation of pH-responsive fluorescent liposome probes. Yao et al. (117) described the production of a pH-responsive fluorescent probe, demonstrating a correlation between the fluorescence signal and the neurological deficit score. In addition, this imaging modality can be used to visualize pathophysiological processes in the vascular system, such as plaque formation and rupture, thrombus formation, and blood flow reduction (118). Compared with traditional tools used in IS diagnosis, such as CT and MRI, fluorescence imaging can diagnose pathological conditions and assess the microenvironment in real-time, thus providing an objective risk assessment of future IS.

The main limitations of fluorescent probes include (1) their limited depth penetration and (2) light interference and autofluorescence. In recent years, with the development of the second near-infrared region, fluorescence imaging has significantly overcome the issues of photon scattering and autofluorescence and allows deep tissue penetration and micrometer spatial resolution (119–121).

#### 3.1.4 Nanotechnology

Nanoparticles (NPs) are colloid carriers with sizes ranging from 1 nm to 1,000 nm. NPs are classified according to their source, distinguishing between synthetic and natural NPs. Synthetic NPs offer a higher level of control over the physicochemical properties of the particles, such as size and surface charge. On the other hand, natural NPs are derived from living cells or formed through biological processes and thus show excellent biocompatibility. Nanotechnology can help overcome the limitations of traditional delivery systems, ranging from large-scale biodistribution to small-scale intracellular delivery. The BBB is widely recognized as a crucial interface between the central nervous system and systemic circulation and poses a significant challenge to the effective delivery of drugs to the brain. NPs are able to cross the BBB through various means, including (1) the induction of local toxic effects that increase the permeability of the BBB, allowing the drug to penetrate the BBB either in its free form or in combination with NPs (122), (2) the promotion of transcytosis by charge adsorption (123), and (3) using receptor-mediated transcytosis based on the presence of specific receptors on the luminal surfaces of the cells (124). Factors such as the size, shape, hydrophobicity, and surface charge of the NPs affect the efficiency of their transmission through the BBB. In general, NPs used for drug delivery to the brain should have specific characteristics, including (1) a negative correlation between the size of the NPs and the permeability of the BBB (125), although this certainly does not mean that smaller is better, as too-small sizes (<10 nm) can be rapidly cleared and excreted by glomerular filtration, so that NPs with diameters of 50–100 nm are usually selected, (2) rod shapes, which have higher adhesion tendencies relative to spherical shapes but are more difficult to prepare (126), and (3) moderate or high negative zeta potentials which are more conducive to brain drug delivery. In general, nanotechnology has brought life back to some drugs that were limited by the BBB permeability problem.

NPs are an exciting development in anatomical and molecular imaging. They have been used in nuclear medicine for many years, where they are generally referred to as colloids, constructed from a carrier platform and an imaging component, such as technetium. The most common radioisotope used for medical imaging is ^99m^Tc, which is ideal for SPECT cameras after an attenuation transition. The applications of SCs for treating IS have been described above, and NPs can provide real-time tracking and visualization of SC processes *in vivo*, specifically, through the coupling of fluorescent dyes and superparamagnetic iron oxide NPs to label SCs and then using MRI to continuously track and identify the colonization, migration, differentiation, and proliferation of the transplanted SCs in IS (127), in addition, the design of NPs with the specific ability to induce SC differentiate in a beneficial manner may be a new therapeutic direction.

In addition to drug delivery and imaging, NPs with different pharmacological activities can be used to treat IS by simultaneously regulating multiple cellular and molecular events produced by inflammation and oxidative stress. Some NPs can use their chemical reducibility to remove oxygen radicals through direct chemical reactions, Yuan et al. (128) constructed a ROS-responsive NP termed the TPCD NP, which showed good antioxidative and anti-inflammatory activities and could be targeted for delivery into the brain. The TPCD NP could significantly inhibit microglia-mediated inflammatory responses and the excessive production of ROS, reduce both neuronal apoptosis and infarct size and improve the prognosis of IS mice. Dong et al. (129) used nano-vesicles from HL-60 cells and loaded Resolvin D2 (a metabolite of DHA with anti-inflammatory and anti-infective activities) into the vesicles. The drug-loaded vesicles bound specifically to the inflamed brain endothelium of IS lesions inhibited endothelial activation and cytokine release and reduced neuroinflammation after IS reperfusion treatment. NPs can also be used as carriers of neuroprotectants, enhancing their BBB penetration and prolonging their half-life to promote neuroprotection. Verma et al. (130) synthesized chitosan-conjugated NIPAAM nanoparticles coated with Tween 80, loaded with the neuroprotective agent riluzole, demonstrating their greatly improved bioavailability and neuroprotective effects even at very low concentrations.

**Figure 3:**
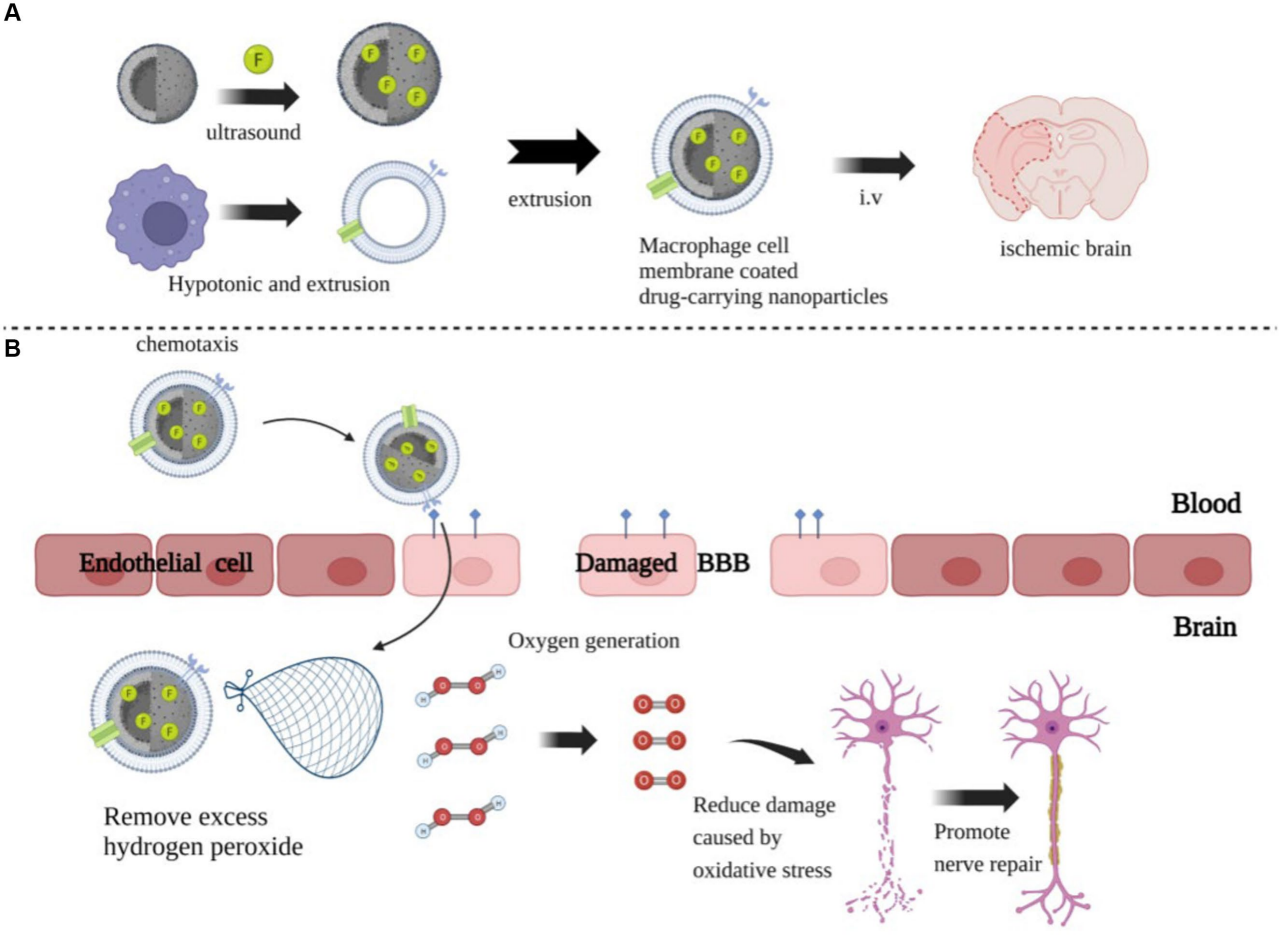
The preparation process and mechanism of action of nanoparticles. **(A)** The preparation of MnO_2_ nanospheres with a honeycomb structure, followed by the addition of the drug FTY, which promotes the polarization of microglial cells into the M2 type, to the MnO_2_ colloid. One-hour ultrasound treatment, followed by centrifugation to remove excess drugs. Next, FTY-loaded MnO_2_ nanospheres were coated with vesicles extracted from rat peritoneal macrophages and injected into the ischemic brain via an intravenous route. **(B)** The coating on the giant cell membrane can increase the specificity of the nanoparticles and reduce their immunogenicity, allowing them to rapidly accumulate in damaged brains. They consume excessive H_2_O_2_ and produce O_2_, reducing ROS accumulation and thereby minimizing neuronal damage and promoting repair.

Due to the complexity of NPs, safety issues such as biocompatibility and immunogenicity deserve attention, and the technology requires continuous improvement. In recent years, the development of biomimetic nanomedical drugs based on living cells or cell membrane vesicles has emerged as a prominent research focus in the field of nanotechnology for the treatment of IS, due to their exceptional biocompatibility, safety, and inherent targeting capabilities. Li et al. (131) developed a macrophage-camouflaged MnO_2_ NP loaded with fingolimod, which could target the ischemic zone by recognizing overexpressed cell adhesion molecules on the damaged vascular endothelium through macrophage membrane proteins (Figure 3A). The MnO_2_ is then released from the NP in acidic lysosomes, allowing it to convert excess H_2_O_2_ to O_2_ and thus improve the hypoxic environment to reduce oxidative stress and neuronal injury (Figure 3B). In addition, the NPs were encapsulated in cell membranes, helping to reduce the immunogenicity of the NPs, prolong their half-life, improve their safety, and to a certain extent make up for the defects of nanotechnology.

### 3.2 Disease mechanism studies

#### 3.2.1 scRNA-seq

The pathological mechanism of IS is not fully understood, and intravenous thrombolysis via rt-PA remains the only effective drug therapy in emergencies (132). However, despite timely and comprehensive treatment, some patients may still experience some degree of functional impairment, such as limb paralysis, aphasia, and skin sensory deficits (133–135). Hence, the search for other potential therapeutic targets for stroke has become an urgent scientific problem. In recent years, people have begun to study the specific signaling pathways involved in IS pathophysiology of IS for the identification of novel therapeutic strategies (136), representing a gradual shift in the focus from the macroscopic to the microscopic level, from studying individual organisms to organs, followed by tissues, and finally to the cellular level. Understanding the survival and gene expression of various cell types during the onset and progression of stroke is crucial. However, the roles of the different subsets of cells in neurovascular units and immune cells during a stroke are difficult to analyze. This has been resolved by the development of scRNA-seq, which can provide a panoramic view of the composition, distribution, and function of cell subpopulations in the microenvironment, while at the same time analyzing the differences in gene expression between cells with the same phenotype, and thus elucidating the molecular mechanisms involved in the disease. scRNA-seq has been widely used in many fields, such as tumor biology, developmental biology, and animal science.

Currently, the application of scRNA—seq in research on IS has been limited but based on previous research in other areas, it holds significant potential for advancing IS research. Paik et al. (137) performed a large-scale scRNA-seq analysis to address the transcriptional heterogeneity of human-induced pluripotent stem cell-derived endothelial cells (iPSC-ECs, a useful tool for cardiovascular disease research), optimizing the differentiation protocol of iPSC-ECs and classifying iPSC-ECs with specific biological functions and identities. This suggests that scRNA-seq can be used to compare different cell subpopulations and cell states in IS and analyze their gene expression patterns. In Zheng’s et al. (138) study, scRNA-seq was used to determine the functions of specific cell subpopulations in various pathways in MCAO mouse models. Moreover, differences in the differentiation of microglia and the potential trajectory branching of macrophage subpopulations were also identified. This study reveals precise transcriptional changes during neuroinflammation at the single-cell level, opening up new avenues for exploring the mechanisms underlying stroke pathogenesis and drug discovery based on cell subtype-specific molecules. Baron et al. (139) used scRNA-seq to reveal dynamic gene expression during the maturation of hematopoietic stem cells (HSCs) and the formation of intra-aortic hematopoietic clusters (IAHCs), by modifying the genes and transcription factor networks activated during the endothelial to-hematopoietic transition (EHT) and IAHCs, such as HSC maturation. Similarly, regulation of gene expression in different cell subsets during IS may be a new therapeutic strategy.

scRNA-seq has emerged as an invaluable tool for single-cell research, enabling the joint analysis of genomics, transcriptomics, epigenomics, proteomics, and metabolomics. Compared with traditional sequencing methods, scRNA-seq has a greater ability to identify new genes without prior knowledge of their sequences, as well as showing higher sensitivity in the detection of rare mutations and the quantification of transcriptomes (140). Additionally, scRNA-seq circumvents biases introduced by polymerase chain reaction (PCR) amplification, as it measures the true abundance of preamplified nucleic acid sequences rather than the relative abundance of the amplified products. However, scRNA-seq also has some limitations, one of which is the occurrence of an “artificial transcriptional stress response,” in which tissue dissociation can induce the expression of stress-related genes, leading to artificial changes in the cellular transcriptomic landscape. Additionally, scRNA-seq can be costly, with the price for sequencing a single sample ranging from $3,000 to $4,000. Therefore, several key factors must be considered when designing scRNA-seq experiments and workflows to ensure the acquisition of accurate and reliable data from each experiment.

#### 3.2.2 Spatial transcriptomics

While scRNA-seq is associated with significant convenience in studying the molecules and cells of the central nervous system, it requires the dissociation of tissue samples, thus losing information about the interrelationships between cells and the space in which the cells are located. The use of spatial transcriptomic (ST) methods can bypass tissue dissociation and preserve this spatial information, thus allowing the assessment of gene expression in thousands of cells in the context of tissue structure. This spatially resolved RNA sequencing can provide information on the relationships between spatial organization and function, which is critical for studying dysregulated cell networks and markers of disease (141). ST technology can be divided into sequence-based ST (sST) and image-based ST (iST). sST techniques typically involve the construction of a spatially indexed surface where each pixel contains a DNA primer with a barcode that provides a unique marker of the location of the pixel in two-dimensional space, then placing the tissue on top of the surface and bringing the resident mRNA into contact with the primer either by the diffusion of RNA from the tissue to the surface or by diffusion of the barcoded primer into the tissue, allowing the capture of the entire mRNA transcriptome. In iST, fluorescent probes specifically label RNA molecules by complementary hybridization, followed by imaging of the probes using fluorescence microscopy. To overcome spectral limitations, new iST techniques typically use multiple rounds of continuous imaging for transcript detection. That is, after signal amplification at the original position of the RNA molecule, different labeling probes are used for multiple labeling imaging. After the collection of multiple rounds of image data, the complete ST data can be generated for the spatial localization of mRNA using spot detection, image registration, and decoding (142).

The concept of spatial transcriptomics was first proposed in 2016 (143). In just a few years of development, ST has been widely applied in various fields, such as tumor biology, neuroscience, and developmental biology (144–151). While its direct application in studying the mechanisms underlying IS pathophysiology has been relatively limited, previous work using ST has successfully created maps of the nervous system, linking brain cell types with functional aspects, such as morphology, physiology, and connectivity, thus providing new insights for research on IS and other brain disorders. Winkler et al. (152) analyzed 181,388 single-cell transcripts derived from adult cerebrovascular cells using single-cell sequencing and ST, followed by the proposal of a cell resolution atlas, allowing the identification of pathological molecular changes in the endothelia associated with arteriovenous malformation (AVM, a major cause of stroke in young people). It was suggested that AVM-induced hemorrhagic apoplexy may be related to the interaction between immune cells and blood vessels, enhancing the understanding of the pathological development of human cerebrovascular diseases and the search for therapeutic targets.

ST has been the focus of research in various fields in recent years. Its development has enhanced the application and scope of transcriptome sequencing. However, current ST methods face challenges such as resolution, sensitivity, and throughput, hindering the precise understanding of normal and abnormal tissues. Overcoming these limitations requires further technological innovation. In addition to improving and optimizing the current universality of ST, we also envision the integration of multiple omics disciplines, such as epigenomics, proteomics, and metabolomics, to elucidate the complexities of cell–cell interactions and their association with disease. Furthermore, in addition to advances in ST technology, further innovations in data analysis strategies are needed for better understanding and interpretation of the data.

#### 3.2.3 3D bioprinting

**Figure 4:**
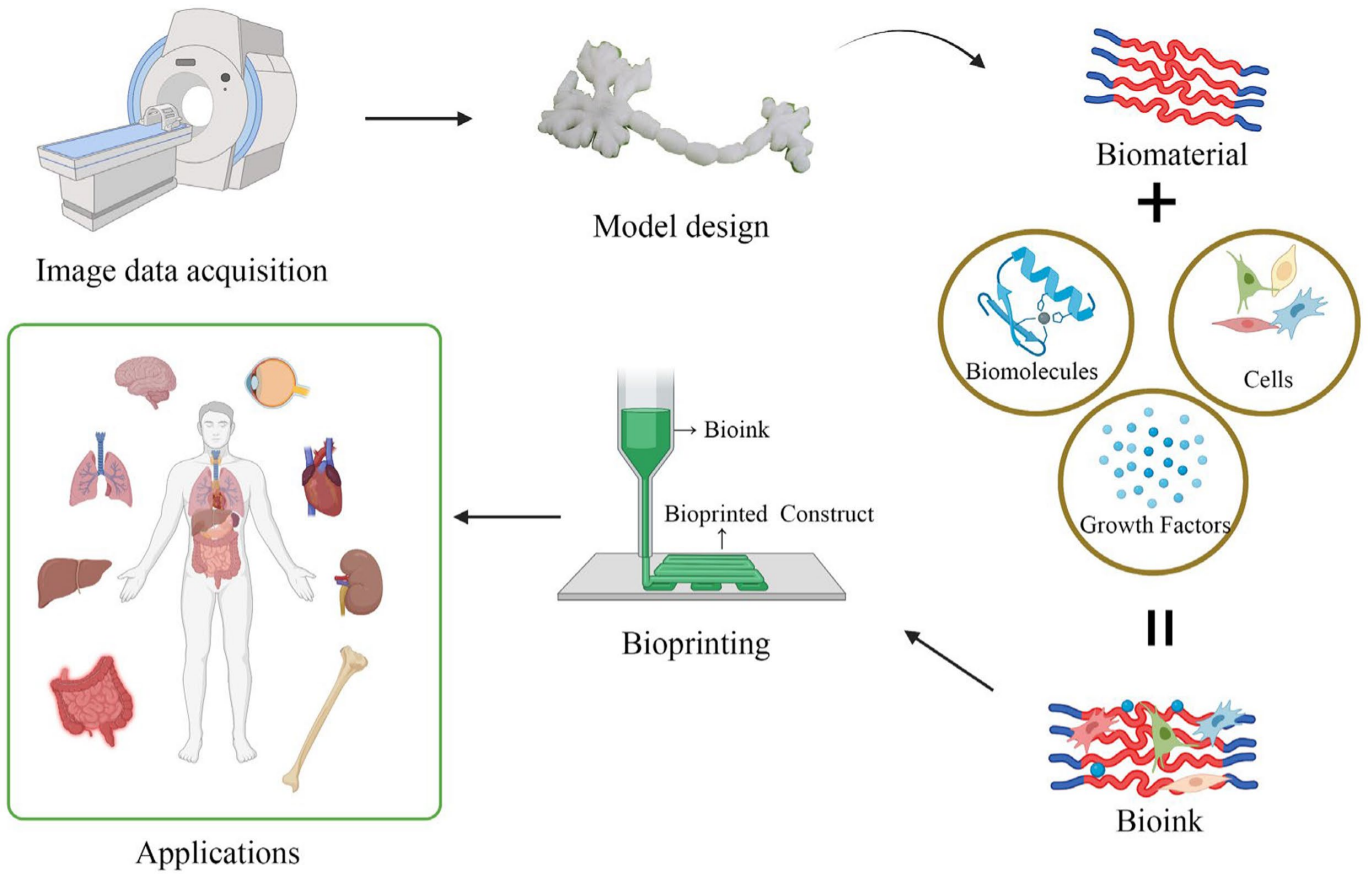
The brief process flowchart of 3D bioprinting. First, obtain imaging data, then use computer assistance to create or acquire the digital model of the biological structure to be printed. Next, prepare the printing materials, such as biological ink or a biological scaffold. Biological ink typically consists of live cells, biomaterials, and growth factors. A biological scaffold primarily functions to provide cell support and a structure for growth. Then, set the printing parameters, such as path, print speed, thickness, etc. After setting the parameters, load the biological ink into the printer’s nozzle, and position it on the target location through the layer-by-layer stacking process. Finally, solidify the material to ensure the stability of the printed structure.

3D bioprinting has undergone rapid development in recent years, making it a groundbreaking innovation in the field of regenerative medicine and biomedical products. Initially, 3D printing was used primarily for creating inanimate objects, such as orthotics for limb rehabilitation in stroke patients and surgical models for educational purposes (153). As technology advanced, the development of tissue engineering scaffolds and organ printing based on 3D printing have emerged. It is also one of the most vibrant and promising directions in 3D printing technology. The implementation of 3D printing typically involves several key steps, namely, the acquisition of image data, the design of 3D models, the selection of appropriate printing materials, and the construction of tissue structures layer-by-layer (LBL) (154, 155). If the printing process involves living cells, it is commonly referred to as bioprinting and the printed structures are referred to as constructs, while if no living cells are involved, the process is simply termed printing, and the printed structures are described as scaffolds. To be more precise, bioprinting is the distribution of small units of cells and biological materials to form tissue-like structures with micrometer precision. Such cells, matrix structural materials, and other necessary biological materials are called “bioinks,” and are then printed on the substrate in a LBL manner, which can be used to produce functional tissue structures and organs (Figure 4). 3D printing, in contrast, does not use cells or biological agents and is commonly used to produce cell-seeded porous polymer scaffolds, prostheses, implants, and surgical models.

As described earlier, 3D-printed engineered scaffolds can be effectively combined with stem cell transplantation, as they provide proper cell location, cell adhesion, and extracellular matrix deposition, while also allowing the transport of gases, essential nutrients, and control factors to promote cell proliferation, survival, and functional differentiation (156). At the same time, because the scaffold also provides physical support, it can also be used to stabilize atherosclerotic plaque, correct oscillations and low shear stress of the endothelia, and prevent plaque formation, and can thus be used as an emerging tool for IS prevention (89). 3D bioprinting involving living cells is currently the major focus. The printing of living cells enables the reconstruction of tissue and organ structures, as well as the restoration of specific biological functions. Revascularization forms the basis of tissue regeneration and is the prerequisite for the clinical treatment of cardiovascular and cerebrovascular diseases and organ repair. Early vascularization relies largely on endothelial cells for the construction of artificial blood vessels *in vitro*, although the network structure formed cannot provide effective perfusion. 3D bioprinting can establish functional vascular networks in various ways. For example, Zou et al. (157) used polyvinyl alcohol (PVA) as a sacrificed material to print sacrificed scaffolds using a bioprinting nozzle, and mixed human umbilical vein endothelial cells (HUVECs) with sodium alginate, agarose solution, and platelet-rich plasma (PRP) as the bioink. After dissolving the sacrificed stent, a flexible, hollow, and microfluid-like vascular structure was formed. The structure was found to be effective in terms of the maintenance time of patency and the network function of the blood vessel. Gao et al. (158) used a coaxial nozzle to extricate partially crosslinked hollow alginate filaments loaded with fibroblasts and smooth muscle cells, followed by printing along a rotating rod template, and inoculation of endothelial cells into the inner wall. The fusion of the adjacent hollow filaments formed two-level fluid channels (macrochannels for mechanical stimulation and microchannels for nutrient transport and chemical stimulation). This 3D hydrogel-based vessel showed high stability, as well as good adaptability and functionality in the vascular circulatory system. Effective vascularization is a prerequisite for the survival of artificial organs. 3D bioprinting is effective in this respect, providing higher biocompatibility and longer service life compared with traditional 3D printing. 3D bio-printed artificial organs and *in vitro* disease models have also raised *in vitro* research to a new height, as traditional *in vitro* research mainly involves *in vitro* cell experiments, representing only a two-dimensional level simulation, which does not effectively reflect the actual situation of diseased tissues and organs in the body and the microenvironment. The stroke model produced by 3D bioprinting is better able to simulate cell-to-cell, protein–protein, and cell-to-protein interactions. This model allows the acquisition of more accurate and reliable data, including information on the spatial distribution of cells an protein expression profiles that are closer to physiological conditions. At the same time, the construction of artificial blood vessels based on 3D bioprinting increases the availability of artificial organs, because effective vascularization can mitigate the necrosis of artificial organs and disease models *in vitro*. In addition, the establishment of an effective artificial neurovascular unit model can assist the investigation into the pathological mechanisms involved in IS, as well as the screening and development of new drugs, thus representing a very attractive application prospect.

Indeed, while bioprinting technology has shown great potential in the laboratory, it still has a long way to go before widespread clinical applications become a reality. To facilitate the transition of bioprinting from the laboratory to the clinical environment, several challenges need to be addressed. One outstanding challenge is the choice of the right printing material. Some scaffolds require both high modulus to maintain mechanical stability and low elastic modulus to simulate the extracellular matrix of nerve cells to provide mechanical stimulation. To solve this problem, Wang et al. (159) designed a 3D vascular structure model using 3D coaxial printing, in which matrix materials containing cells were injected into the vascular structure and solidified by ultraviolet irradiation to provide physical support. Therefore, low-modulus matrix materials can be used directly for printing without considering the stability of the support. In addition, the addition of Pluronic F127 sacrificial materials during coaxial printing and their removal after preparation may allow the realization of the adjustable porosity of blood vessels (larger porosity is more conducive to neuronal differentiation). This study not only solved the contradictory problem of the selection of matrix material but also improved the feasibility of the application of the artificial neurovascular unit model. In future research, continued innovation and collaboration between scientists, engineers, regulators, and clinicians will be essential to overcome these challenges and unlock the full potential of bioprinting for medical applications.

#### 3.2.4 16S rRNA sequencing

In bacteria, 5S, 16S, and 23S ribosomal RNAs are organized in a gene cluster and expressed as a single operon. The sizes, sequences, and secondary structures of these three rRNA genes are highly conserved in different bacterial species. In particular, 16S rRNA, which is present in all bacterial chromosomes and genomes, contains 9 highly variable and species-specific regions (V1–V9), flanking highly conserved constant regions (160). Due to its modest size of about 1,500 bp, it can not only reflect the differences between different bacterial genera but also provide sequence information. Therefore, 16S rRNA sequencing is considered the gold standard for microbiome analysis and classification (161), and is widely used to characterize bacterial communities.

The human gut accommodates trillions of bacteria that play crucial roles in various physiological processes and are vital for the overall maintenance of human health. Studies have demonstrated that stroke can lead to alterations in the composition and function of the gut microbiota, resulting in gut microbiota dysbiosis. This dysbiosis, in turn, worsens cerebral infarction, suggesting a potential bidirectional relationship between gut microbiota dysbiosis and stroke (162). However, the precise nature of the interaction between stroke and the microbiome remains largely unexplored. The application of 16S rRNA sequencing can analyze the composition of gut bacterial communities in patients with IS, and differences in community compositions may be linked to different pathogenic pathways associated with IS. For example, specific flora have been suggested to be involved in the formation of atherosclerosis and inflammation (163–165). In addition, 16S rRNA sequencing can be combined with non-targeted metabolomics to cluster different flora and bacterial metabolites, determining the associations between microorganisms and metabolites, and thus revealing the impact of microbial metabolites on host physiological functions, as well as the response of host immune system to microorganisms, which is conducive to timely detection of potential risks of IS. In addition, microbe-metabolite association pairs can be used as potential targets for future *in vivo*/*in vitro* experiments (166, 167). In short, 16S rRNA sequencing is an important aspect of IS research, which can reveal the information related to microbial composition and the interaction between host and microorganism, provide a theoretical basis and research direction for the pathogenesis of IS, and assist in the understanding of the occurrence and development of IS.

The development of high-throughput sequencing and metagenomics has led to an understanding of the physiological and pathological relationships between the intestinal flora and the brain. However, there are still many problems associated with the use of targeting the intestinal flora for treating IS, one of which is due to technical defects. Other researchers consider that the role of intestinal flora should not be over-emphasized, as this, with the current level of technology, may result in erroneous conclusions. Nevertheless, it is undeniable that an in-depth exploration of the interaction between intestinal flora and human health can deepen our understanding of IS and provide a broader scope for its target treatment.

**Figure 5:**
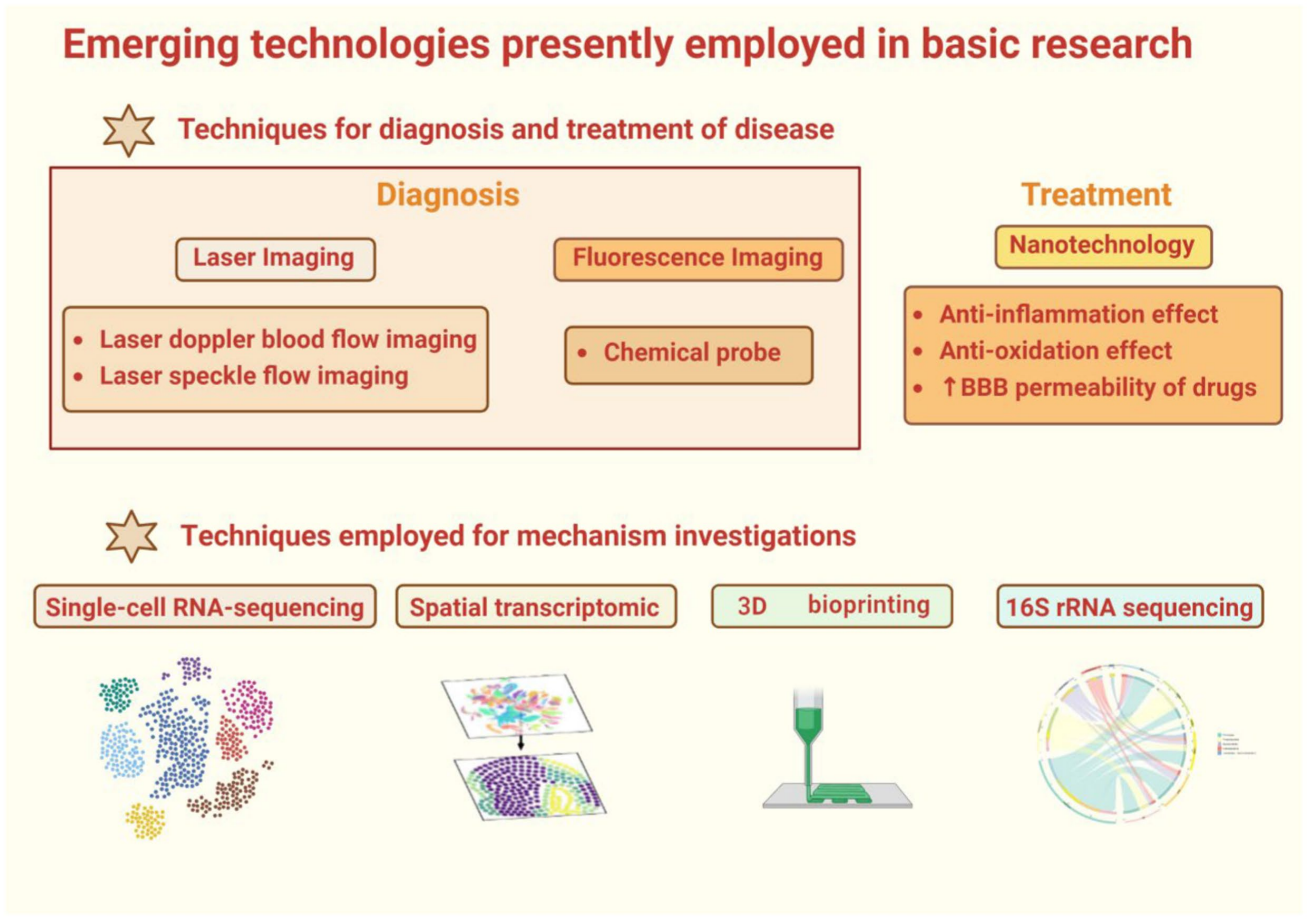
This is a graphical summary containing the emerging technologies covered in this review that are currently used in basic research. The main emerging basic research technologies are divided into two categories, one for the diagnosis and treatment of diseases, and the other for the study of disease mechanisms. Among them, there are two main imaging techniques used to diagnose diseases. One is laser imaging, including laser Doppler imaging and laser speckle imaging. The other is fluorescence imaging. The techniques used to treat diseases are mainly introduced in nanotechnology. In the second part, the technologies for disease mechanism research include single-cell RNA sequencing, spatial transcriptome, 16S rRNA sequencing and 3D bioprinting.

To assist readers in comprehending the basic technologies involved in this review, refer to Figure 5.

## 4 Summary and outlook

Currently, stroke continues to pose a significant burden (168). Therefore, it is crucial to develop more effective prevention strategies and timely diagnostic methods to mitigate the adverse impact of this disease. Medical imaging remains indispensable for the diagnosis of stroke, augmenting the ability of physicians to assess the condition and extending the treatment window for patients. Advances in machine learning have given rise to decision-support tools, enhancing the interpretability of these studies. In the future, neuroimaging technologies may attain greater precision in the location of stroke lesions, thereby offering enhanced support for personalized treatments. Regenerative medicine, involving techniques such as cell therapy, tissue engineering, and the use of biomaterials, seeks to facilitate tissue repair and regeneration. In the context of IS, the application of stem cell therapy and the use of growth factors has garnered considerable attention but is still in its nascent stages. Further progress hinges on breakthroughs in policies and regulations, financial investments, product development, and collaboration among various stakeholders. Future research should delve deeper into the biological compatibility and safety of stem cells and tissue engineering scaffolds. Nanomedicine presents an exciting prospect in the field of IS. This interdisciplinary field combines nanotechnology and medicine, offering new possibilities for treating IS. Nanoparticles can be designed for drug delivery as they are able to cross the blood-brain barrier, allowing precise release of therapeutic substances. Additionally, nanotechnology contributes to improving the resolution of imaging techniques. In the future, further research in nanomedicine will require more clinical trials and validation to ensure its safety and efficacy. The development of sequencing technologies contributes to advancing the research on mechanisms related to IS, and together with target identification and nanoparticle techniques, it is of significant importance for future drug development in the field of stroke research.

## Author contributions

QC: Writing – original draft, Conceptualization. SZ: Writing – review & editing. WL: Supervision, Writing – review & editing. XiaoS: Supervision, Writing – review & editing. YL: Funding acquisition, Supervision, Writing – review & editing. XiaoboS: Funding acquisition, Supervision, Writing – review & editing.
